# ARL11 correlates with the immunosuppression and poor prognosis in breast cancer: A comprehensive bioinformatics analysis of ARL family members

**DOI:** 10.1371/journal.pone.0274757

**Published:** 2022-11-11

**Authors:** Ning Xie, Qiuai Shu, Ziwei Wang, Xindi Huang, Yalan Wang, Bin Qin, Yan Chen, Na Liu, Lei Dong, Jian Wu, Yahua Song

**Affiliations:** 1 Department of Gastroenterology, The Second Affiliated Hospital of Xi’an Jiaotong University, Shaanxi, China; 2 Shaanxi Key Laboratory of Gastrointestinal Motility Disorders, Xi’an Jiaotong University, Shaanxi, China; 3 Department of Oncology, Xijing Hospital, The Fourth Military Medical University, Xi’an, Shaanxi Province, China; 4 Xijing Hospital of Digestive Diseases, Xijing Hospital, The Fourth Military Medical University, Xi’an, Shaanxi, China; Central University of Rajasthan, INDIA

## Abstract

ADP-ribosylation factor-like protein (ARL) family members (ARLs) may regulate the malignant phenotypes of cancer cells. However, relevant studies on ARLs in breast cancer (BC) are limited. In this research, the expression profiles, genetic variations, and prognostic values of ARLs in BC have been systematically analyzed for the first time using various databases. We find that ARLs are significantly dysregulated in BC according to the TCGA database, which may result from DNA methylation and copy number alteration. Prognostic analysis suggests that ARL11 is the most significant prognostic indicator for BC, and higher ARL11 predicts worse clinical outcomes for BC patients. Further functional enrichment analysis demonstrates that ARL11 enhances the immunosuppression in BC, and dysregulation of ARL11 is significantly associated with immune infiltration in various types of cancer. Our results demonstrate the potential of ARL11 as an immune therapeutic target for BC.

## Introduction

Breast cancer is the leading cause of cancer deaths in women [1]. Hence, there is an urgent need to understand the molecular mechanisms that underlie the tumorigenesis and progression of BC, in order to pave the way for the development of novel biomarkers with predictive and therapeutic potential for BC. The ADP-ribosylation factor (ARF) family members of small GTP-binding (G) proteins belong to the Ras superfamily and include the ARFs, SAR1, Tripartite Motif-containing protein 23 (TRIM23), and ARF-like (ARL) proteins. ARLs, which structurally resemble other ARF family members, have more than 20 types in humans and are integral in controlling membrane traffic, vesicular transport, cytoskeleton organization, and cell migration via a cyclic switch between GTP-bound state (active) and GDP-bound state (inactive) [2]. Moreover, dysregulation of ARLs has enormous effects on various diseases, including cancer [3].

Mounting evidence suggests that aberrant expression of ARLs contributes greatly to the tumorigenesis of various types of tumors, including hepatocellular carcinoma, osteosarcoma, and colorectal cancer [4–10]. For example, decreased expression of ARL2 markedly suppresses cervical cancer cell proliferation, migration, and invasion [8]. In addition, ARL8B is pivotal for the 3D invasive growth of prostate cancer cells *in vitro* and *in vivo* [11]. Downregulation of ARL4C can even suppress AKT signaling and decrease cell proliferation in lung adenocarcinoma cells [12], and ARL13B is conducive to the progression of gastric cancer *in vitro* and *in vivo* by modulating smoothened trafficking and activating Hedgehog signaling [13]. Finally, ARL11 is also implicated in a number of familial cancer types [14–16]. All of this indicates the promise of ARLs as therapeutic targets for tumors. However, our existing knowledge is limited on the prognostic value and biomedical functions of ARLs in BC.

In this paper we investigated the expression profiles, genetic alterations, and prognostic values of ARLs in BC, and we identified ARL11 as a vital prognostic indicator for invasive BC. Higher expression of ARL11 was correlated with adverse clinical outcomes of BC patients. Furthermore, enrichment analysis indicated that ARL11 might also significantly boost immunosuppression for BC. Our results highlight the specific role of ARL11 in BC, which may assist in the future development of novel precision therapeutics and biomarkers for BC.

## Methods

### UALCAN database analysis

UALCAN is a comprehensive, user-friendly, and interactive web-portal that executes the analysis of The Cancer Genome Atlas (TCGA) data (http://ualcan.path.uab.edu/index.html) [17]. We used this database to evaluate mRNA expression levels of ARLs between normal samples and breast cancer and to compare the expression differences among different cancer subtypes. Student’s *t*-test *p*-values less than 0.05 were considered to indicate statistically significant test results.

### cBioPortal database analysis

The cBio Cancer Genomics Portal (cBioPortal) (http://www.cbioportal.org/) imparts visualization tools for more than 5,000 tumor samples from 232 cancer studies in the TCGA database [18–20]. In this paper we used this tool to analyze the Breast Invasive Carcinoma (TCGA, Firehose Legacy, n = 1,108) cohort in particular. The search parameters included mutations, mRNA expression *Z*-scores and putative copy-number alterations from GISTIC. Co-expression analysis was also executed according to online instructions of the cBioPortal.

### Kaplan-Meier plot analysis

Kaplan-Meier plots (http://kmplot.com/analysis/) were engaged to analyze the relationship between the expression of ARL genes and survival rates in breast cancer based on the strength of the hazard ratios (HR) and log-rank *p*-values [20, 21].

### MEXPRESS tool analysis

The MEXPRESS tool (https://mexpress.be), a user-friendly tool for visualizing and interpreting TCGA data, contains information on mRNA expression, DNA methylation, clinical data, and the relationships between these objects [22]. For this paper we used the MEXPRESS tool to evaluate the impact of methylation status on the expression levels of ARL3, ARL4C, ARL4D, and ARL11 in BC.

### The Cancer Regulome tools and data analysis

We also employed the Cancer Regulome tools and data (http://explorer.cancerregulome.org/) from the TCGA database in order to create circos plots to show the genomic location of ARL11 and its related-genes in BC. Spearman correlation was utilized to reveal the pairwise correlation between genes. The circos plots only display genes with *p*-value <0.01.

### Functional enrichment analysis

For our purposes, we took a Spearman’s correlation coefficient that exceeded 0.15 to indicate a correlation between ARL11 and its co-expressed genes and then used the clusterProfiler to perform GO (Gene Ontology) and KEGG (Kyoto Encyclopedia of Genes and Genomes) enrichment analysis of co-expressed genes that were correlated with ARL11. We then employed Metascape online software (http://metascape.org) to frame the interaction network of enrichment terms. All analysis was implemented with default software parameters [23].

### TIMER 2.0 database analysis

The Tumor Immune Estimation Resource 2.0 (TIMER 2.0) (http://timer.comp-genomics.org/) is a computational tool for the methodical analysis of the correlation between the target gene expression and the tumor-infiltrating immune cells of 32 cancer types [24, 25]. In this research, we used TIMER 2.0 to analyze the relationship between ARL11 expression and immune infiltration in pan-cancer.

## Results

### The expression profiles of ARLs in BC

**Fig 1:**
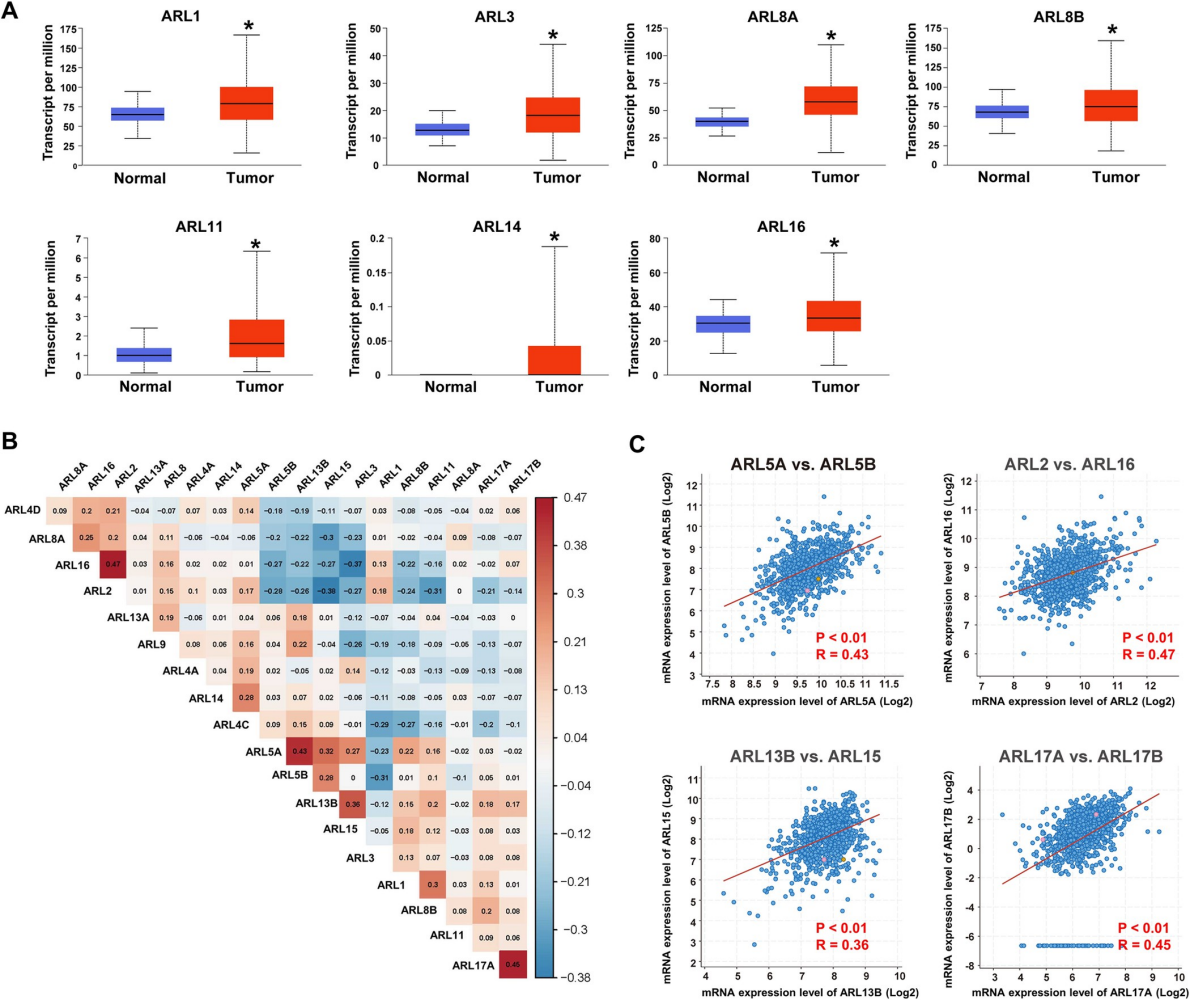
Expression profiles of ARLs in BC. **(A)** Box plots show the differential expression of ARLs between BC and adjacent normal tissues based on the TCGA dataset analyzed using the UALCAN database (*p < 0.05). (**B**) Spearman correlation analysis shows the expression correlations of ARLs in BC. (**C**) The scatter plots show the Spearman correlation between the expression of ARL5A and ARL5B, ARL13B and ARL15, ARL16 and ARL2, and ARL17A and ARL17B.

We first explored the expression differences in ARLs between BC and normal samples using the UALCAN database. We found that 18 ARLs were remarkably dysregulated in BC. As shown in **Fig 1A**, ARL1, ARL3, ARL8A, ARL8B, ARL11, ARL14, and ARL16 were significantly upregulated in BC tissues compared to normal tissues (*p* < 0.05). Additionally, ARL2, ARL4A, ARL4C, ARL4D, ARL6, ARL9, ARL10, ARL13B, and ARL17B were significantly downregulated in BC (*p* < 0.05) (**S1 Fig**). We then analyzed the co-expression status among ARLs in BC based on the cBioPortal database and performed Spearman correlation analysis using the expression profile data (RNA Seq V2 RSEM) of ARLs from the cBioPortal. As shown in **Fig 1B**, many positive correlations were identified between ARL5A and ARL5B (R = 0.43, *p* < 0.01), ARL2 and ARL16 (R = 0.47, *p* < 0.01), ARL13B and ARL15 (R = 0.36, *p* < 0.01), and ARL17A and ARL17B (R = 0.45, *p* < 0.01) (**Fig 1C**).

Furthermore, we evaluated the expression status of 18 ARLs in different subtypes of BC including luminal, HER2-positive, and triple-negative BC (TNBC), and the results showed that expression status differed significantly across different BC subtypes. As shown in **S2 Fig**, ARL2, ARL10, ARL13B, and ARL17B were markedly downregulated in HER2-positive BC compared to the luminal and triple-negative subtypes. Additionally, ARL9 and ARL16 were upregulated in TNBC, and ARL4D and ARL15 were significantly downregulated in TNBC compared to the luminal and HER2-positive subtypes.

We also evaluated the expression status of ARLs in the TIMER 2.0 database and found 18 ARLs significantly dysregulated in BC. Moreover, the expression status of most ARLs was consistent between the two independent datasets. The detailed information for *p*-values is shown in **S1 and S2 Tables.**

### Genetic alterations of ARLs in BC

**Fig 2:**
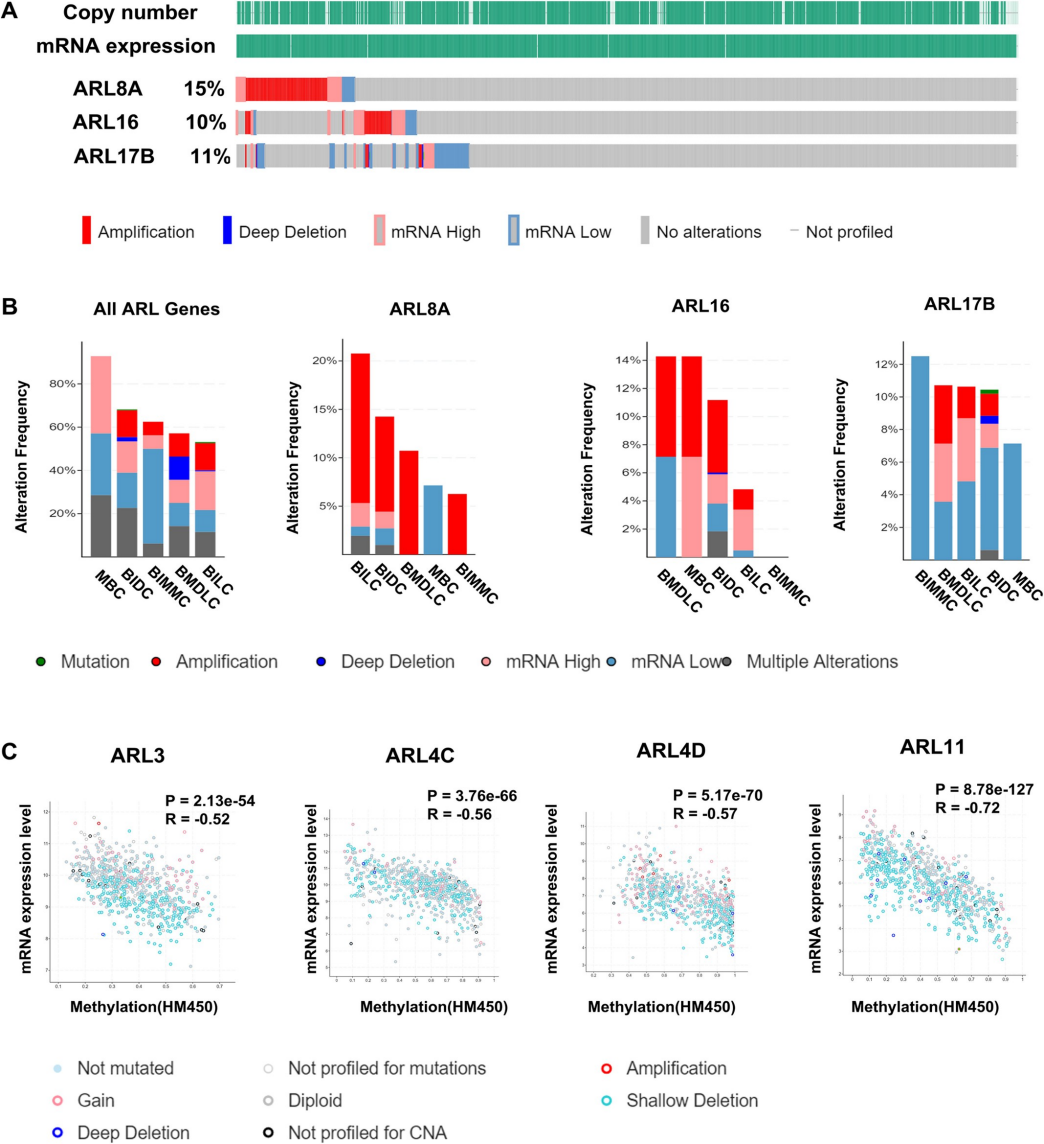
Genetic alterations of ARLs in BC. **(A)** The OncoPrint visual summaries of genetic variations of ARL8A, ARL16, and ARL17B in the TCGA database. **(B)** Alteration frequencies of ARLs (ARL8A, ARL16, and ARL17B) in different BC subtypes, including mutations, amplification, deep deletion, mRNA dysregulation, and multiple changes. **BMDLC**: Breast Mixed Ductal and Lobular Carcinoma; **BILC**: Breast Invasive Lobular Carcinoma; **BIDC**: Breast Invasive Ductal Carcinoma. **MBC**: Metaplastic Breast Cancer. **BIMMC**: Breast Invasive Mixed Mucinous Carcinoma **(C)** Association of DNA methylation with ARLs (ARL3, ARL4C, ARL4D, and ARL11) mRNA expression in BC.

In order to acquire a comprehensive understanding of ARLs in BC, we analyzed the genetic alterations of ARLs using the cBioPortal. We found diverse degrees of genetic variations in the 22 ARLs, ranging from 2.6% to 15%, among which ARL8A had the highest mutation ratio (15%) (**Fig 2A** and **S3 Fig**). The mutation ratios of ARL16 and ARL17B were comparatively higher, up to 10% and 11%, respectively (**Fig 2A**). In addition, the modification frequencies of all ARLs as well as ARLs with higher mutation rates (mutation ratio ≥ 10%) in different subtypes of BC are shown in **Fig 2B**. We also found copy number amplification (CNA) for most ARLs was in several different subtypes of BC but not for metaplastic breast cancer (MBC). Furthermore, we employed Spearman’s correlation analysis to explore the relationship between the status of promoter DNA methylation and levels of ARLs mRNA expression. Interestingly, as shown in **S3 Table**, there was negative correlation between mRNA expression and DNA methylation for most ARLs, and the correlation coefficients between mRNA expression and DNA methylation for ARL3, ARL4C, ARL4D, and ARL11 were the highest (R ≥ 0.5, *p* < 0.05) (**Fig 2C**). Therefore, we also performed specific methylation site analysis of ARL3, ARL4C, ARL4D, and ARL11 with the MEXPRESS dataset (**Table 1**).

**Table 1:** DNA methylation sites of ARL3, ARL4C, ARL4D, and ARL11.

Genes	Methylation sites	Position	Pearson-r	*p*-value
**ARL3**	cg06361405	3’UTR	0.337	3.08E-21
	cg24435571	3’UTR	-0.175	2.24E-07
	cg10992686	gene body	-0.484	2.89E-34
	cg05024762	gene body	-0.128	0.000148
	cg25355065	gene body	-0.177	1.53E-07
	cg10961055	gene body	-0.071	3.46E-02
	cg08216291	TSS ± 200	-0.081	1.63E-02
	cg15302128	TSS ± 200	-0.126	1.79 E-04
**ARL4C**	cg24441922	3’UTR, 1st exon	-0.159	1.57E-07
	cg05204104	3’UTR, 1st exon	-0.52	8.13E-40
	cg15016771	3’UTR, 1st exon	-0.417	1.41E-30
	cg11509907	1st exon	-0.42	1.06E-29
	cg21650900	1st exon	-0.184	5.94E-08
	cg21460828	1st exon	-0.19	1.97E-08
	cg04942334	5’UTR, 1st exon	-0.123	0.000319
	cg13539030	TSS ± 1500	-0.154	6.64E-06
	cg09453076	TSS ± 1500	-0.51	7.26E-37
	cg05308656	TSS ± 1500	-0.494	1.04E-35
	cg15235893	TSS ± 1500	-0.408	2.53E-29
	cg09935994	TSS ± 1500	-0.284	9.16E-18
**ARL4D**	cg01065373	TSS ± 1500	-0.114	3.08E-21
	cg23635580	TSS ± 1500	-0.0723	2.24E-07
	cg12842219	TSS ± 200	-0.0722	2.89E-34
	cg02136628	5’UTR, 1st exon	-0.138	1.48E-04
	cg04309025	5’UTR	-0.137	1.53E-07
	cg19252218	gene body	-0.448	3.46E-02
	cg27147556	gene body	-0.547	1.63E-02
	cg18256949	gene body	-0.448	1.79E-04
	cg06795960	3’UTR	-0.416	3.08E-21
	cg14545159	chr17:41479473	-0.181	2.24E-07
**ARL11**	cg11765205	TSS ± 200	-0.562	5.04E-43
	cg08772163	TSS ± 200	-0.35	1.86E-22
	cg11827082	TSS ± 200	-0.486	3.04E-36
	cg01425731	5’UTR, 1st exon	-0.669	6.00E-51
	cg04638679	gene body	-0.676	9.43E-51

**Fig 3:**
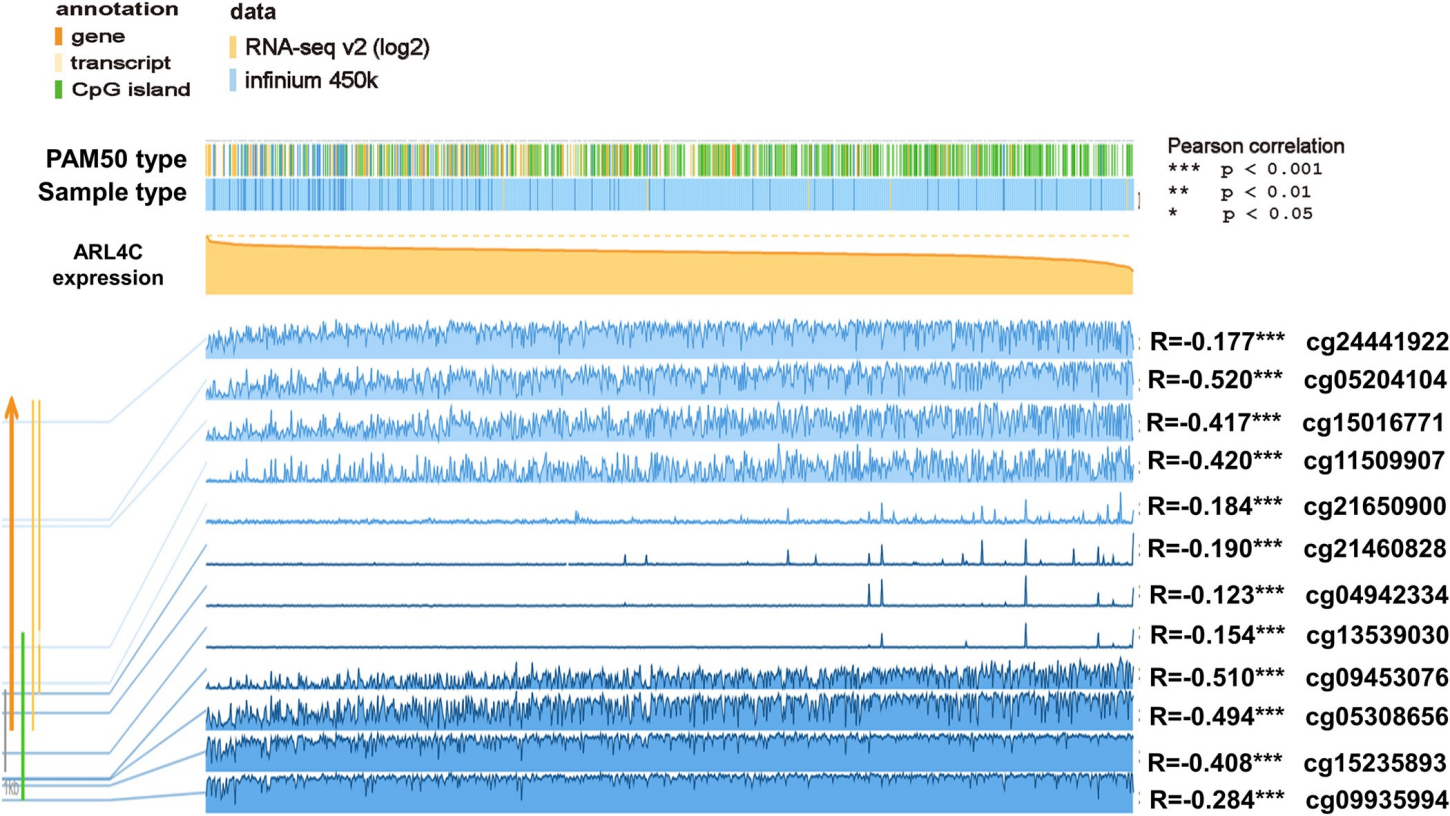
Visualization of the correlation between ARL4C expression and DNA methylation in the TCGA-BRCA cohort (n = 871) using the MEXPRESS online database. There are 12 DNA methylation sites that mays negatively regulate the expression level of ARL4C (correlation coefficients ranged from -0.123 to-0.510, *p* < 0.001).

Intriguingly, our results indicated that methylation status in different sites might regulate the expression level of ARL3. For instance, ARL3 expression was negatively correlated with the DNA methylation status (correlation coefficient ranged from -0.071 to -0.484, *p* < 0.001). Nevertheless, methylation of cg06361405 at 3’UTR was positively associated with ARL3 expression (R = +0.337, *p* < 0.01). Additionally, we found a significant inverse association between ARL4C, ARL4D, and ARL11 expression and DNA methylation at all genomic regions (correlation coefficients ranged from -0.123 to -0.494, *p* < 0.001) (**Table 1** and **Fig 3**). Moreover, cg01425731 at the 5’-UTR of the first exon was the most significant site of ARL11, and hypomethylation at this site prompted the upregulation of ARL11 in BC (R = -0.669, *p* < 0.001). Collectively, our results indicate that CNA and DNA methylation may feature prominently in the genetic regulation of most ARLs.

### The prognostic value of different ARLs in BC

**Fig 4:**
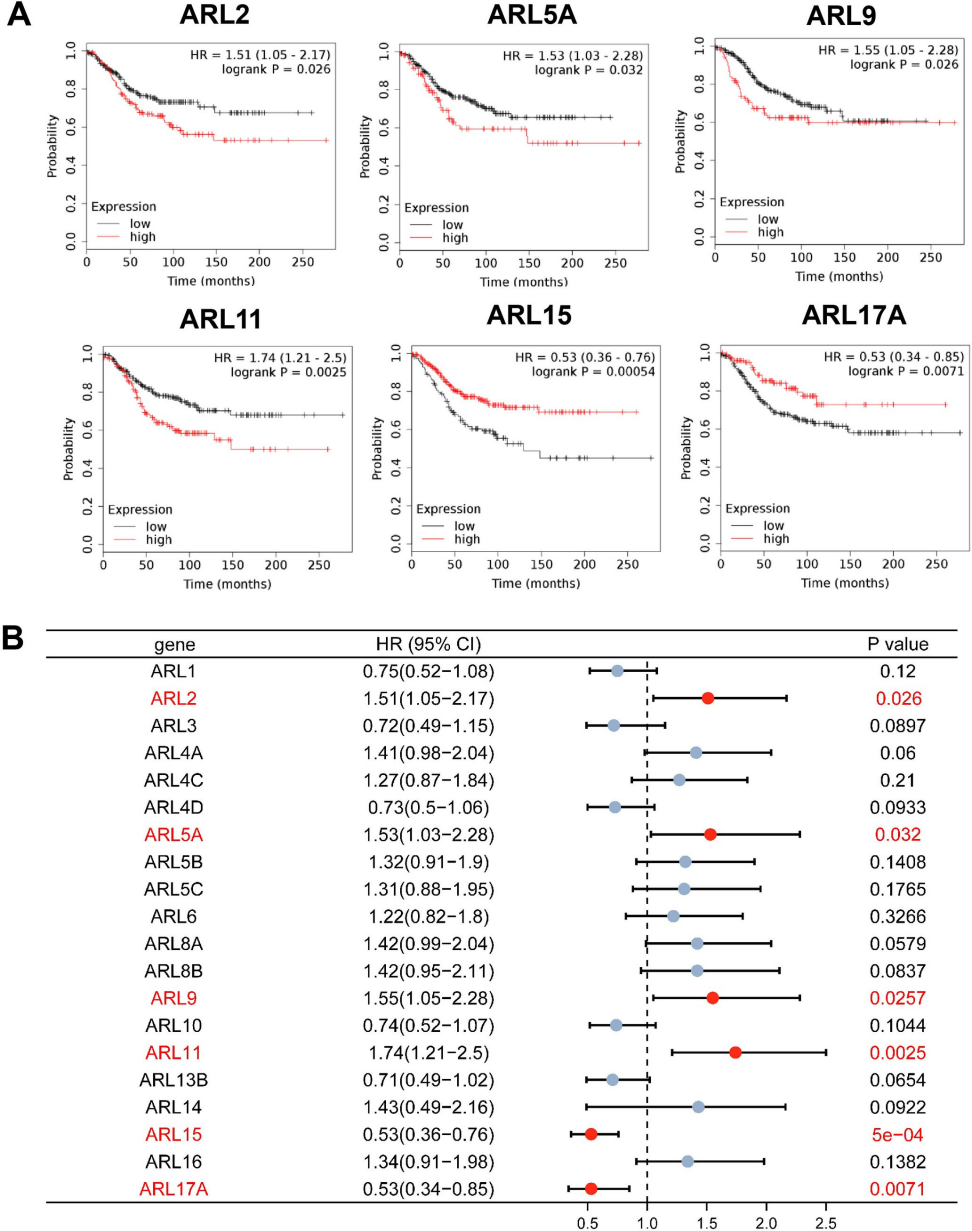
Prognostic value of ARLs in BC. **(A)** Survival analysis of ARLs in BC (RFS in Kaplan–Meier plotter, *p<0.05). Higher expression levels of ARL2, ARL5A, ARL9, and ARL11 indicate worse RFS (HR>1), whereas higher expression levels of ARL15 and ARL17A had noteworthy ties to better RFS (HR1). **(B)** The forest plot shows the distribution of hazard ratios across ARLs in BC from the Kaplan-Meier plotter.

We further explored the prognostic value of different ARLs in BC patients based on the E-MTAB-365 cohort by using the Kaplan-Meier (K-M) plotter to inquire into the effects of ARLs on the recurrence-free survival (RFS) of BC patients. As displayed in **Fig 4A and 4B**, this analysis revealed a remarkable association between higher expression levels of ARL2, ARL5A, ARL9, and ARL11 and worse RFS, whereas higher expression levels of ARL15 and ARL17A had noteworthy ties to better RFS. These results, without indications of statistical significance are shown in **S4 Fig**.

Additionally, we also analyzed the effects of ARLs on the overall survival (OS) of BC patients in the TCGA dataset using the TIMER 2.0 tool. As shown in **S5 Fig**, overexpression of ARL11 was significantly related to worse OS for BC patients. Also of note, ARL11 was the most prominent clinical indicator among ARLs for BC patients after systemic evaluation of their expression status and prognostic value.

### Correlation between mRNA level of ARL11 and immunosuppression in BC

**Fig 5:**
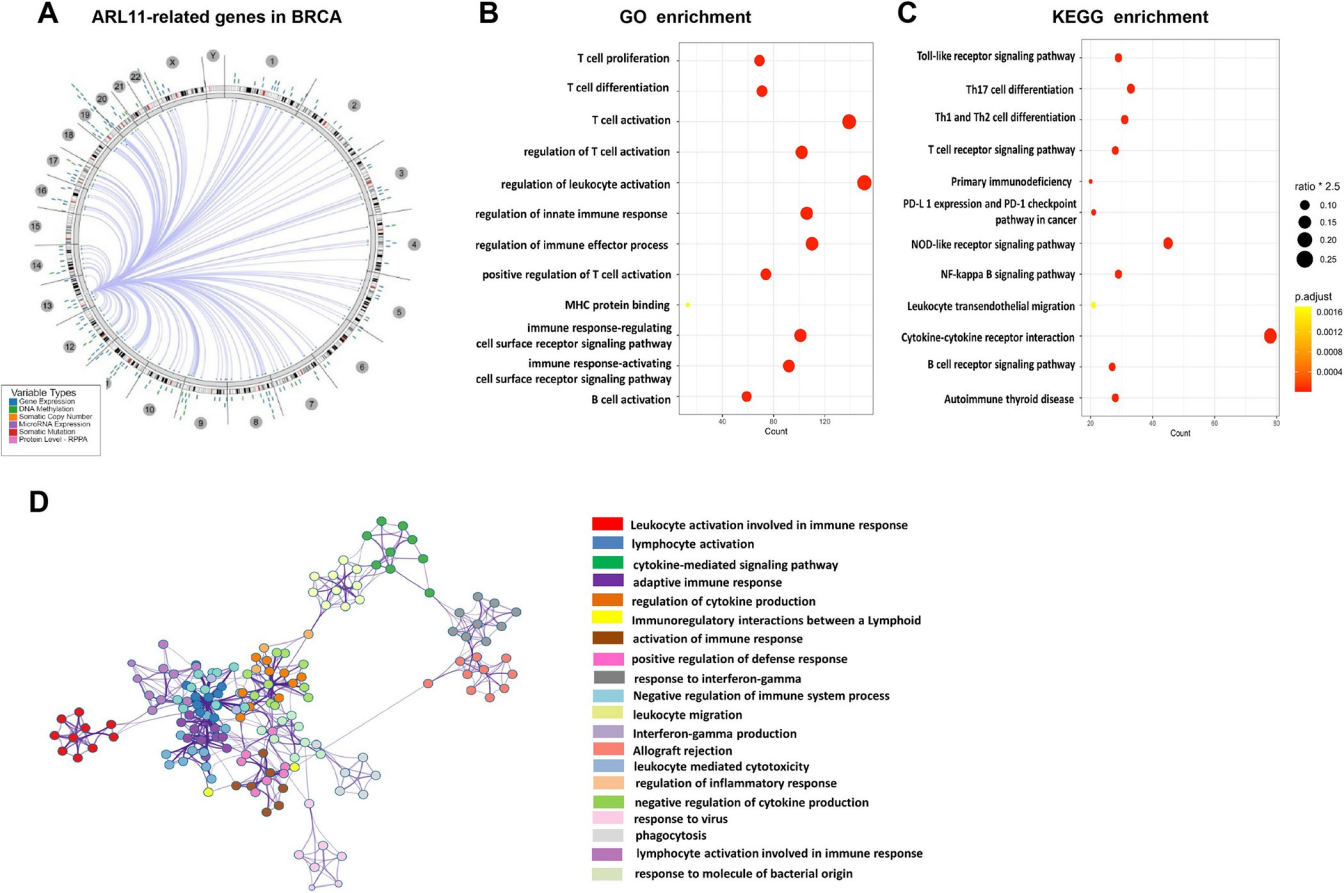
Functional enrichment analysis of ARL11-associated genes in BC. **(A)** A circos plot shows the correlation between ARL11 and other BC genes. **(B)** A bubble plot of the GO enrichment analysis colored by *p*-value. **(C)** A bubble plot of the KEGG enrichment analysis colored by *p*-value. **(D)** A network of the top 20 enriched terms colored by clusters.

To unravel the mechanisms underlying the prognostic value of ARL11 in BC, we analyzed the possible molecular functions of ARL11 based on the TCGA database. The circos plot in **Fig 5A** shows the genomic location of ARL11 and all ARL11-related genes in BC. As displayed in **Fig 5B and 5C**, GO (Gene Ontology) and KEGG (Kyoto Encyclopedia of Genes and Genomes) enrichment analysis indicated that ARL11-associated genes were significantly linked to immunosuppressing processes such as T cell differentiation (gene ratio = 71/1130, *p* = 5.22E-30), PD-L1 expression and the PD-1 checkpoint pathway in cancer (gene ratio = 21/659, *p* = 7.22E-06), the and Toll-like receptor signaling pathway (gene ratio = 29/659, *p* = 2.28E-09). To identify the internal associations of these processes, we filed the top 20 clusters of GO and KEGG as a network plot using Metascape online tools in which we deemed a Kappa similarity > 0.3 to indicate a connection (**Fig 5D**). In addition, we investigated the relationship between ARL11 expression and immune infiltration in pan-cancer and found that dysregulation of ARL11 was significantly associated with immune infiltration in various cancer types (**S4 Table**). Notably, the correlation between ARL11 expression and immune infiltration varied across different subtypes of BC. In brief, function enrichment analysis and correlation analysis indicated that the prognostic value of ARL11 may result from its role in enhancing tumor immunosuppression.

## Discussion

The above findings have demonstrated that ARLs are involved in cancer progression. Nevertheless, the clinical relevance of ARLs in BC and their potential mechanisms are not fully understood. To this end, we examine the role of ARLs in BC using bioinformatics analysis. Interestingly, co-expression analysis showed high correlations among ARLs in BC. A number of studies have indicated the existence of collaboration between small GTP protein family members in regulating tumor progression [26–28], and our bioinformatics analysis further demonstrates that ARLs may indeed function collaboratively in BC.

In accordance with the expression status of ER, PR and HER2, BC can be categorized into four subtypes: luminal A (ER^+^ or PR^+^/HER2^-^), luminal B (ER^+^ or PR^+^/HER2^+^), HER2 positive (HER2^+^), and triple-negative BC (TNBC, ER^-^/PR^-^/HER2^-^) [29]. Using the UALCAN online tool, we evaluated the expression levels of ARLs in BC and normal tissues, and the results indicate that the expression levels of several ARLs vary significantly across different subtypes of BC. Therefore, our results provide evidence that the expression profiles of ARLs are BC subtypes specific.

Our genetic analysis showed that genetic alterations, including CNA and DNA methylation status, are engaged in dysregulation of ARLs in BC. We found that DNA methylation status conversely relates to the mRNA expression of several ARLs in BC, such as ARL3, ARL4C, ARL4D, and ARL11 (R ≥ 0.5, *p* < 0.05). Multiple sites of ARL3, ARL4C, ARL4D, and ARL11 genes were found to be hypomethylated in BC patients. DNA methylation is widely recognized as a major epigenetic regulation involved in different stages of tumorigenesis and cancer development. The hypomethylation at the cg24441922 site in particular has been found to contribute to the dysregulation of ARL4C in lung cancer [30], and we found the same result for BC. In short, we found correlation between the DNA methylation status and dysregulation and oncogenic functions of most ARLs in BC.

Considering the oncogenic impact of ARLs, we further performed the prognostic analysis of 22 ARLs in BC, and found that6 ARLs might serve as potential prognostic biomarkers for BC and that ARL11 is the most significant prognostic indicator for BC. ARL11 was initially identified as a low-penetrance cancer gene at the chromosome 13q14.3 that gets continually deleted in some hematopoietic and solid tumors [31, 32]. Researchers have demonstrated that ARL11 variants may contribute to the familial risk of various cancer types, such as chronic lymphocytic leukemia, melanoma, breast cancer, prostate cancer, colorectal cancer, and ovarian cancer [16, 33–36]. More importantly, a current study has found that ARL11 is highly expressed in several kinds of immune cells and may also serve as a positive regulator of extracellular signal-regulated kinase (ERK) signaling in macrophages [37]. However, the specific mechanisms of ARL11 in BC remain poorly understood. Our enrichment analysis demonstrated that ARL11 expression is involved in several immunosuppressing processes in BC, including T cell differentiation and PD-L1 expression and the PD-1 checkpoint pathway in cancer. As tumor immunosuppression is a critical step of preinvasive-to-invasive transition and relates to poor prognoses in BC [38], we speculate that the prognostic role of ARL11 in BC may be due to its function in boosting tumor immunosuppression.

## Conclusion

This is the first study to date to characterize the expression patterns, genetic alterations, and prognostic value of ARLs in BC. More importantly, this study finds ARL11 to be a vital prognostic indicator for BC; higher ARL11 expression predicts a worse prognosis. Further functional analysis showed that ARL11’s prognostic role may result from its promotion of tumor immunosuppression. This study provides new insight into the exact role of ARL11 in BC and emphasizes its potential role as an innovative predictive biomarker and therapeutic target for BC patients.

## Supporting information

S1 Fig Expression status of ARLs which are downregulated in BC compared with normal tissues with statistical significance (*p* < 0.01). (TIF)

S2 Fig Expression alterations of ARLs in different BC subtypes. (TIF)

S3 Fig The OncoPrint summarized the genetic variations of ARLs in BC based on cBioportal. (TIF)

S4 Fig Recurrence free survival analysis of ARLs by Kaplan–Meier plotter without statistical significance. (TIF)

S5 Fig The forest plot showed the overall survival analysis of ARLs based on TIMER 2.0 database (*p* ≥ 0.05). (TIF)

S1 Table P-value of differential expression of ARLs between BC and normal samples using UALCAN and TIMER 2.0 database. (PDF)

S2 Table P-value of differential expression of ARLs among different subtypes of BC. (PDF)

S3 Table The Spearman’s correlation analysis between DNA methylation and ARLs’ mRNA expression in cBioportal database. (PDF)

S4 Table The correlation between ARL11 expression and immune infiltration in pan-cancer. (PDF)
