# Supplementary figures and images for: ARL11 correlates with the immunosuppression and poor prognosis in breast cancer: A comprehensive bioinformatics analysis of ARL family members
Source: PLoS One. 2022 Nov 11;17(11):e0274757. doi: 10.1371/journal.pone.0274757 (PMC9651578; doi:10.1371/journal.pone.0274757)

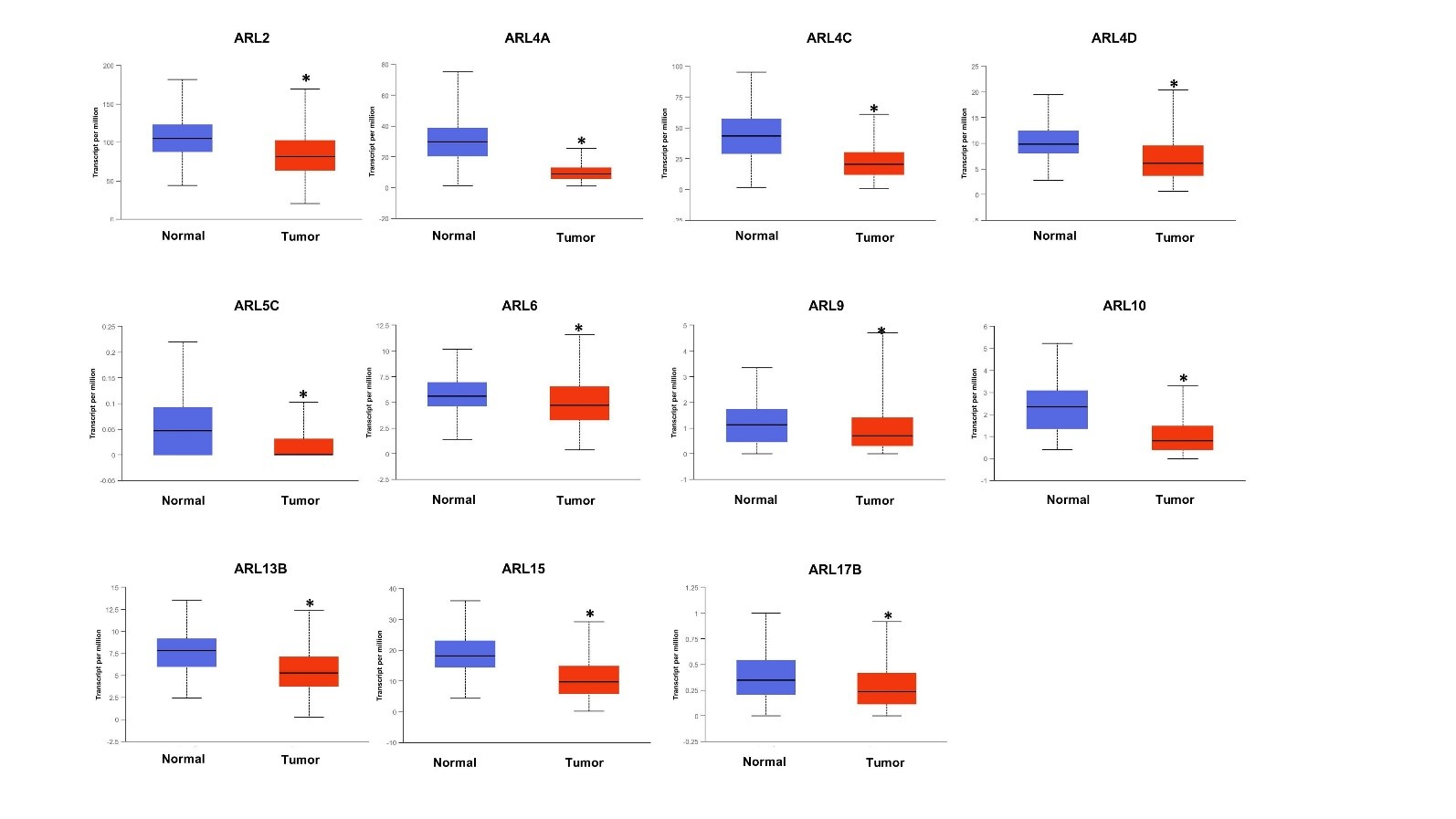

Supplement: S1 Fig — (TIF) [file pone.0274757.s001.tif]

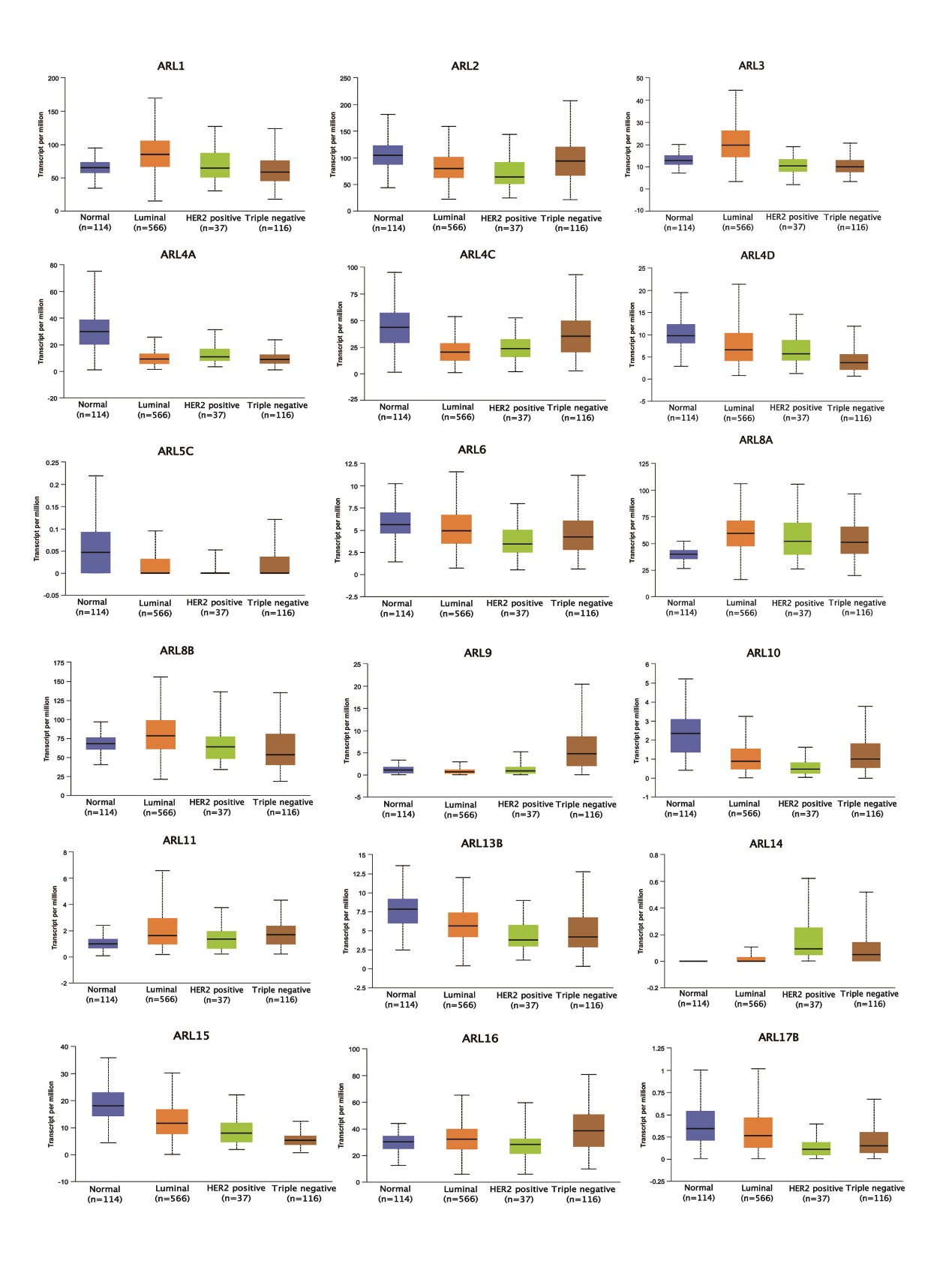

Supplement: S2 Fig — (TIF) [file pone.0274757.s002.tif]

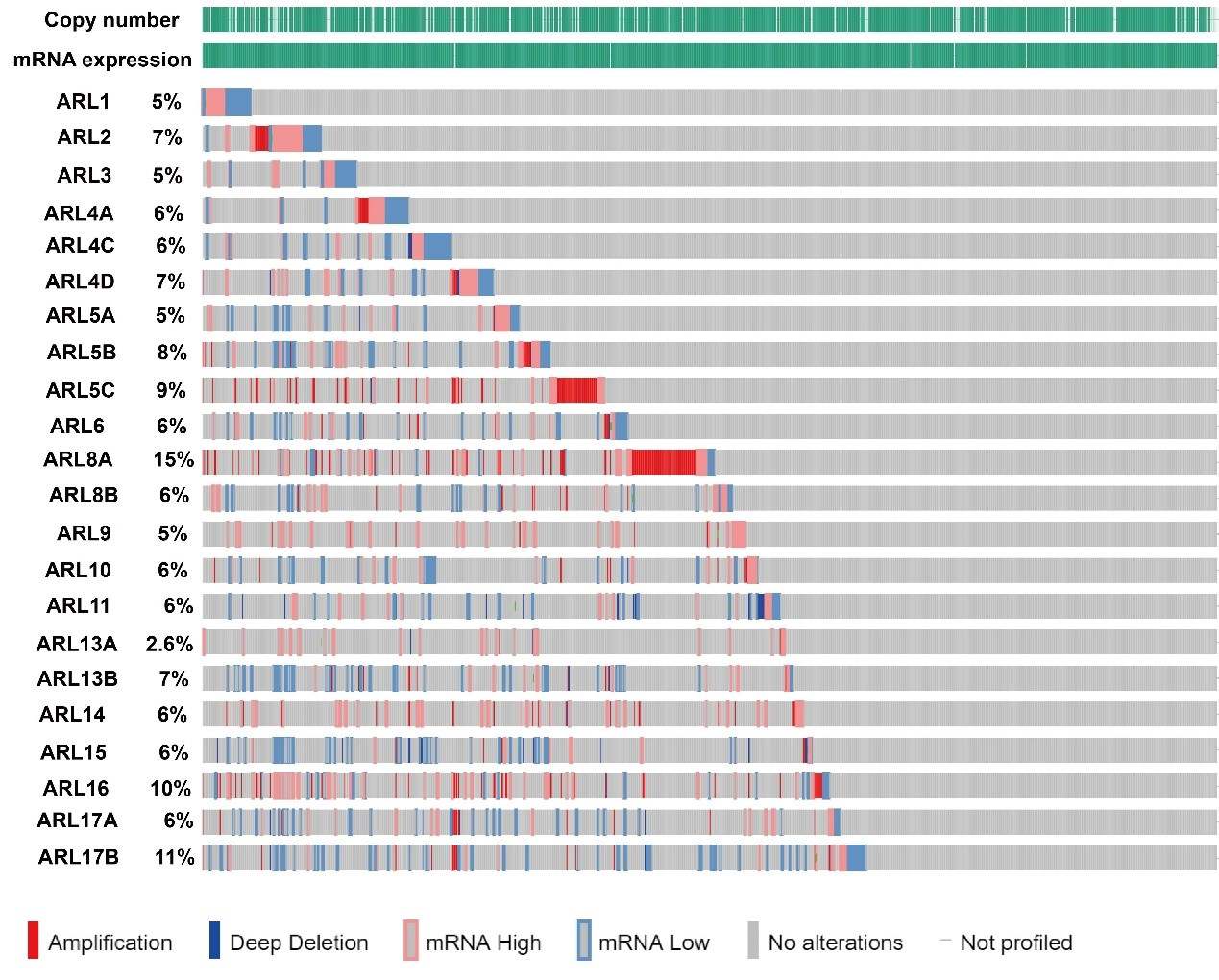

Supplement: S3 Fig — (TIF) [file pone.0274757.s003.tif]

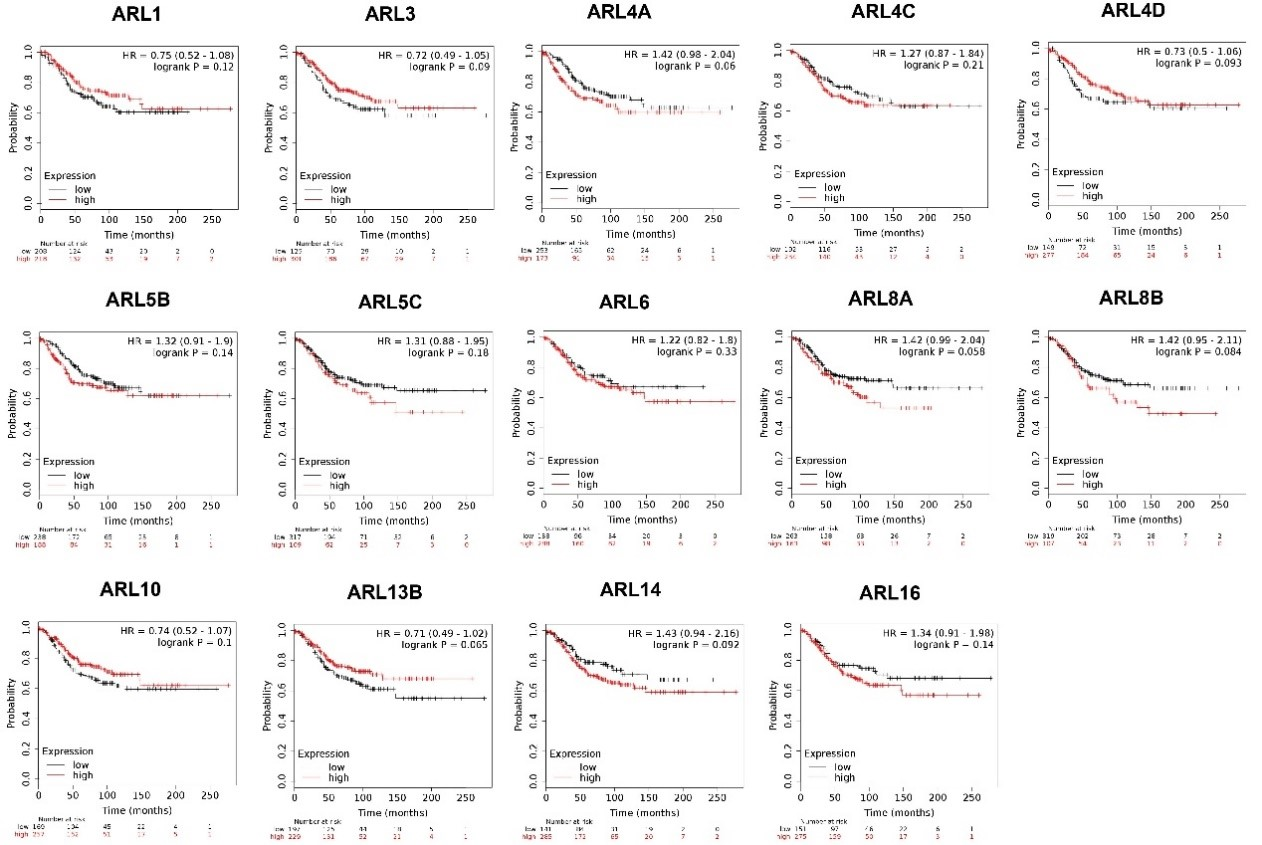

Supplement: S4 Fig — (TIF) [file pone.0274757.s004.tif]

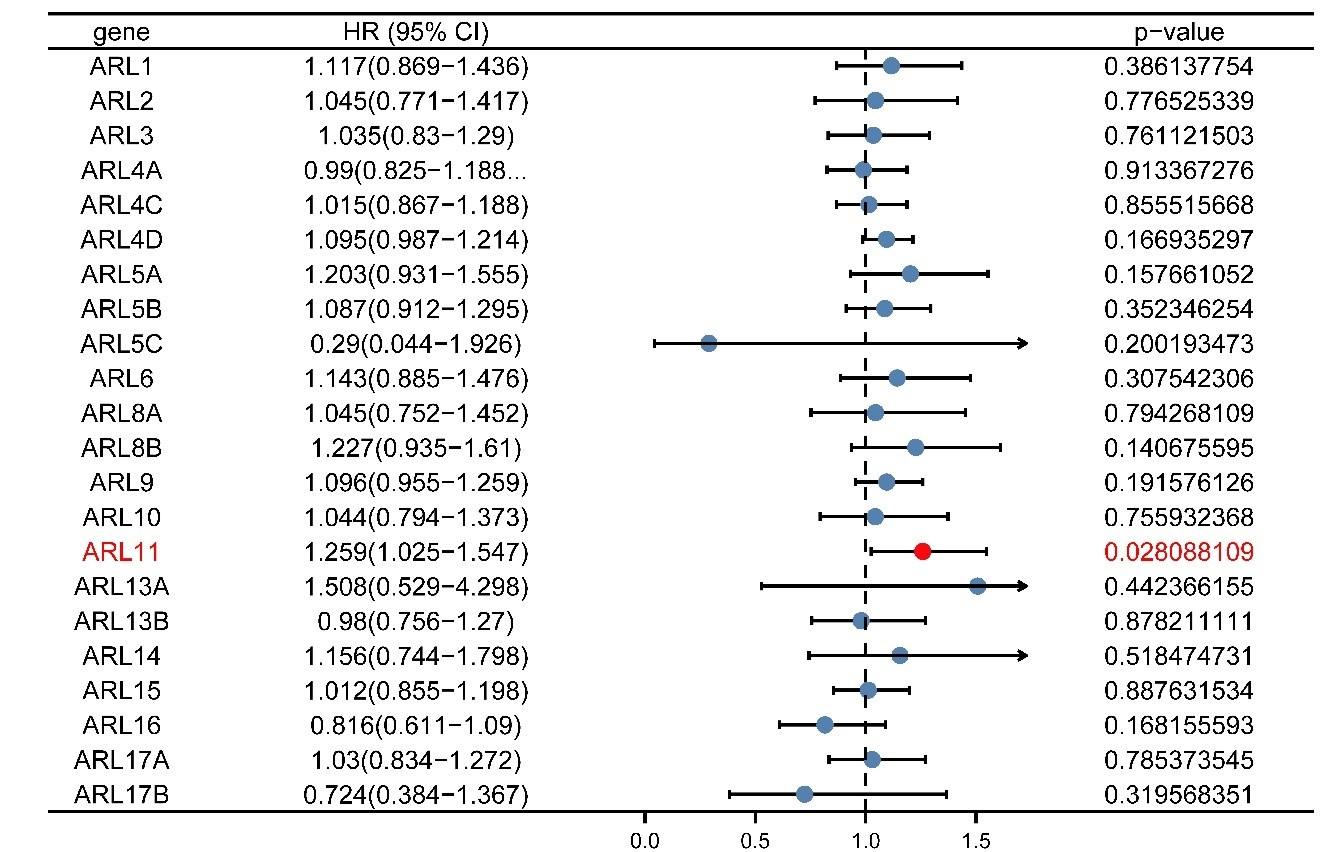

Supplement: S5 Fig — (TIF) [file pone.0274757.s005.tif]
