# Supplementary material for: ARL11 correlates with the immunosuppression and poor prognosis in breast cancer: A comprehensive bioinformatics analysis of ARL family members
Source: PLoS One. 2022 Nov 11;17(11):e0274757. doi: 10.1371/journal.pone.0274757 (PMC9651578; doi:10.1371/journal.pone.0274757)
Supplement: S1 Table — (PDF) [file pone.0274757.s006.pdf]

S1 Table. P-value of differential expression of ARLs between BC and normal samples using UALCAN and TIMER 2.0 database.

**Genes**	**P-value in UALCAN database**	**P-value in TIMER 2.0 database**
ARL1	<1E-12(up)	5.73E-11(up)
ARL2	1.60E-08(down)	7.75E-12(down)
ARL3	<1E-12(up)	1.79E-13(up)
ARL4A	<1E-12(down)	1.06E-49(down)
ARL4C	<1E-12(down)	4.93E-18(down)
ARL4D	1.01E-04(down)	6.72E-13(down)
ARL5A	6.67E-01(down)	1.45E-01(down)
ARL5B	4.66E-01(down)	3.88E-01(down)
ARL5C	1.10E-03(down)	9.97E-13(down)
ARL6	1.92E-02(down)	1.12E-03(down)
ARL8A	<1E-12(up)	6.41E-38(up)
ARL8B	2.33E-15(up)	3.24E-05(up)
ARL9	1.41E-10(down)	8.52E-01(down)
ARL10	<1E-12(down)	1.26E-24(down)
ARL11	<1E-12(up)	1.79E-13(up)
ARL13A	9.91E-01(down)	3.29E-03(down)
ARL13B	9.15E-12(down)	5.24E-12(down)
ARL14	1.37E-09(up)	1.40E-10(up)
ARL15	<1E-12(down)	5.74E-23(down)
ARL16	<1E-12(up)	1.66E-05(up)
ARL17A	6.72E-02(down)	7.04E-01(down)
ARL17B	1.44E-04(down)	2.46E-05(down)
