# Supplementary material for: ARL11 correlates with the immunosuppression and poor prognosis in breast cancer: A comprehensive bioinformatics analysis of ARL family members
Source: PLoS One. 2022 Nov 11;17(11):e0274757. doi: 10.1371/journal.pone.0274757 (PMC9651578; doi:10.1371/journal.pone.0274757)
Supplement: S2 Table — (PDF) [file pone.0274757.s007.pdf]

S2 Table. P-value of differential expression of ARLs among different subtypes of BC.

genes	Normal-vs-Luminal	Normal-vs-HER2 Positive	Normal-vs-TNBC	Luminal-vs-HER2 Positive	Luminal-vs-TNBC	Positive-vs-TNBC
**ARL1**	<1E-12	1.56E-01	6.09E-01	1.44E-03	1.62E-12	7.88E-02
**ARL2**	1.07E-09	1.08E-08	2.18E-01	3.39E-02	7.40E-04	2.23E-05
**ARL3**	<1E-12	8.20E-02	1.57E-02	1.83E-12	<1E-12	9.20E-01
**ARL4A**	1.62E-12	1.63E-12	1.62E-12	2.33E-02	3.54E-01	2.99E-01
**ARL4C**	1.62E-12	5.49E-09	8.56E-01	5.39E-01	1.09E-07	1.75E-05
**ARL4D**	3.04E-04	2.13E-01	3.19E-04	9.12E-01	4.86E-02	3.25E-01
**ARL5C**	8.98E-04	4.24E-01	3.22E-04	8.32E-01	6.61E-01	7.35E-01
**ARL6**	2.50E-01	6.19E-05	6.70E-03	4.02E-03	2.87E-02	1.57E-01
**ARL8A**	<1E-12	1.82E-05	2.03E-12	7.93E-02	2.03E-02	9.03E-01
**ARL8B**	1.62E-12	2.46E-01	1.69E-01	7.26E-02	3.38E-11	6.01E-02
**ARL9**	6.76E-01	1.05E-01	9.76E-10	9.32E-02	7.51E-10	2.00E-04
**ARL10**	1.62E-12	1.62E-11	7.19E-04	9.92E-03	3.50E-02	9.60E-04
**ARL11**	1.62E-12	1.36E-02	1.14E-09	3.62E-03	9.02E-04	3.43E-01
**ARL13B**	7.68E-10	4.55E-09	3.76E-06	9.37E-04	3.05E-01	5.01E-02
**ARL14**	1.52E-05	5.38E-04	8.52E-03	7.32E-03	3.13E-02	3.15E-01
**ARL15**	1.75E-12	2.93E-12	1.62E-12	3.24E-04	<1E-12	1.65E-02
**ARL16**	1.56E-08	2.77E-01	8.57E-09	4.87E-01	1.92E-03	2.96E-02
**ARL17B**	2.86E-03	2.44E-15	5.46E-08	2.24E-14	1.83E-05	5.00E-04
