# Supplementary material for: ARL11 correlates with the immunosuppression and poor prognosis in breast cancer: A comprehensive bioinformatics analysis of ARL family members
Source: PLoS One. 2022 Nov 11;17(11):e0274757. doi: 10.1371/journal.pone.0274757 (PMC9651578; doi:10.1371/journal.pone.0274757)
Supplement: S3 Table — (PDF) [file pone.0274757.s008.pdf]

S3 Table. The Spearman's correlation analysis between DNA methylation and ARLs' mRNA expression in cBioportal database.

Genes	Spearman's correlation coefficient	P-value
ARL1	-0.23	1.44E-10
ARL2	-0.15	1.687E-5
ARL3	-0.52	2.13E-54
ARL4A	-0.42	3.36E-35
ARL4C	-0.56	3.76E-66
ARL4D	-0.57	5.17E-70
ARL5A	-0.26	6.89E-14
ARL5B	-0.20	2.30E-81
ARL5C	None	None
ARL6	-0.21	5.17E-9
ARL8A	-0.15	2.284E-5
ARL8B	-0.30	2.44E-18
ARL9	-0.51	8.45E-54
ARL10	-0.31	3.53E-19
ARL11	-0.72	8.78E-127
ARL13A	None	None
ARL13B	-0.15	1.445E-5
ARL14	-0.08	0.0313
ARL15	-0.32	8.97E-20
ARL16	-0.28	4.67E-16
ARL17A	-0.11	3.754E-3
ARL17B	0.01	0.862
