# Supplementary material for: ARL11 correlates with the immunosuppression and poor prognosis in breast cancer: A comprehensive bioinformatics analysis of ARL family members
Source: PLoS One. 2022 Nov 11;17(11):e0274757. doi: 10.1371/journal.pone.0274757 (PMC9651578; doi:10.1371/journal.pone.0274757)
Supplement: S4 Table — (PDF) [file pone.0274757.s009.pdf]

S4 Table. The correlation between ARL11 expression and immune infiltration in pan-cancer.

cancer	variable	Spearman's correlation coefficient	p
ACC	CD8+ T Cell	0.18554665	0.116036
BLCA	CD8+ T Cell	0.01782554	0.733241
BRCA	CD8+ T Cell	-0.084971849	0.007352
BRCA-Basal	CD8+ T Cell	-0.061102402	0.423169
BRCA-Her2	CD8+ T Cell	-0.223302876	0.059361
BRCA-LumA	CD8+ T Cell	-0.121386071	0.005717
BRCA-LumB	CD8+ T Cell	0.041940282	0.563534
CESC	CD8+ T Cell	0.124167325	0.038903
CHOL	CD8+ T Cell	0.086014098	0.623216
COAD	CD8+ T Cell	-0.21327353	0.000368
DLBC	CD8+ T Cell	0.145795527	0.36306
ESCA	CD8+ T Cell	-0.002268712	0.975887
GBM	CD8+ T Cell	-0.021871691	0.799738
HNSC	CD8+ T Cell	-0.083346974	0.064713
HNSC-HPV-	CD8+ T Cell	-0.051648363	0.302812
HNSC-HPV+	CD8+ T Cell	-0.135522905	0.205406
KICH	CD8+ T Cell	0.056522647	0.654719
KIRC	CD8+ T Cell	0.127729712	0.006027
KIRP	CD8+ T Cell	0.164679656	0.008039
LGG	CD8+ T Cell	-0.06154516	0.179167
LIHC	CD8+ T Cell	-0.005067981	0.925274
LUAD	CD8+ T Cell	-0.082793178	0.06624
LUSC	CD8+ T Cell	0.107349451	0.019018
MESO	CD8+ T Cell	0.135520311	0.216217
OV	CD8+ T Cell	0.119692267	0.059296
PAAD	CD8+ T Cell	0.118726432	0.121951
PCPG	CD8+ T Cell	0.149538884	0.053754
PRAD	CD8+ T Cell	-0.094463431	0.054205
READ	CD8+ T Cell	-0.048775867	0.648007
SARC	CD8+ T Cell	0.097723985	0.127933
SKCM	CD8+ T Cell	0.175051966	0.000169
SKCM-Metastasis	CD8+ T Cell	0.198365083	0.000172
SKCM-Primary	CD8+ T Cell	0.07663583	0.443931
STAD	CD8+ T Cell	0.058578349	0.255284
TGCT	CD8+ T Cell	-0.20167986	0.014302
THCA	CD8+ T Cell	-0.013991037	0.757858
THYM	CD8+ T Cell	-0.027315502	0.771984
UCEC	CD8+ T Cell	0.293117125	0.00558
UCS	CD8+ T Cell	-0.207198055	0.136576
UVM	CD8+ T Cell	0.394963791	0.000378

ACC	CD4+ T Cell	0.147645	0.212562
BLCA	CD4+ T Cell	-0.00892	0.864536
BRCA	CD4+ T Cell	0.083939	0.008103
BRCA-Basal	CD4+ T Cell	0.170771	0.024262
BRCA-Her2	CD4+ T Cell	0.003679	0.97553
BRCA-LumA	CD4+ T Cell	0.047422	0.281814
BRCA-LumB	CD4+ T Cell	0.056047	0.440029
CESC	CD4+ T Cell	0.191877	0.001332
CHOL	CD4+ T Cell	0.122484	0.483332
COAD	CD4+ T Cell	-0.0521	0.389419
DLBC	CD4+ T Cell	0.053272	0.7408
ESCA	CD4+ T Cell	0.008002	0.915097
GBM	CD4+ T Cell	0.12759	0.137333
HNSC	CD4+ T Cell	0.020951	0.642945
HNSC-HPV-	CD4+ T Cell	0.03715	0.458736
HNSC-HPV+	CD4+ T Cell	-0.0394	0.713932
KICH	CD4+ T Cell	NA	NA
KIRC	CD4+ T Cell	0.072822	0.118431
KIRP	CD4+ T Cell	0.111849	0.072899
LGG	CD4+ T Cell	0.033679	0.46258
LIHC	CD4+ T Cell	0.132094	0.014073
LUAD	CD4+ T Cell	0.058997	0.190956
LUSC	CD4+ T Cell	0.090067	0.049307
MESO	CD4+ T Cell	0.074382	0.498689
OV	CD4+ T Cell	0.107687	0.08995
PAAD	CD4+ T Cell	0.167498	0.028542
PCPG	CD4+ T Cell	0.120597	0.12056
PRAD	CD4+ T Cell	0.014336	0.770648
READ	CD4+ T Cell	0.191577	0.070475
SARC	CD4+ T Cell	0.193515	0.002397
SKCM	CD4+ T Cell	0.265402	8.32E-09
SKCM-Metastasis	CD4+ T Cell	0.272884	1.83E-07
SKCM-Primary	CD4+ T Cell	0.133488	0.181047
STAD	CD4+ T Cell	-0.00859	0.867592
TGCT	CD4+ T Cell	0.021102	0.799735
THCA	CD4+ T Cell	0.070559	0.119554
UCEC	CD4+ T Cell	0.056158	0.551096
UCS	CD4+ T Cell	0.159946	0.1366
UVM	CD4+ T Cell	0.15272	0.274954
UCEC	CD4+ T Cell	0.063162	0.585254
ACC	B Cell	0.180767718	0.125902642
BLCA	B Cell	0.050785592	0.331274854
BRCA	B Cell	0.126870414	6.04E-05
BRCA-Basal	B Cell	0.19211099	0.011099422

BRCA-Her2	B Cell	0.021202151	0.85968322
BRCA-LumA	B Cell	0.120284553	0.006175497
BRCA-LumB	B Cell	0.147905154	0.040626173
CESC	B Cell	0.119630204	0.046680351
CHOL	B Cell	0.234053965	0.175966617
COAD	B Cell	0.139178141	0.020957843
DLBC	B Cell	-0.053469384	0.739873547
ESCA	B Cell	-0.096976916	0.195295309
GBM	B Cell	-0.066885842	0.437409162
HNSC	B Cell	-0.02764658	0.54067808
HNSC-HPV-	B Cell	-0.042893978	0.392225339
HNSC-HPV+	B Cell	0.076800548	0.474392781
KICH	B Cell	0.018187217	0.885660943
KIRC	B Cell	0.07258655	0.119631615
KIRP	B Cell	0.247092652	6.02E-05
LGG	B Cell	0.138791841	0.002356199
LIHC	B Cell	0.066169685	0.220224721
LUAD	B Cell	-0.060517656	0.179748794
LUSC	B Cell	-0.012399827	0.787070894
MESO	B Cell	-0.104951009	0.339114124
OV	B Cell	0.065680098	0.301924756
PAAD	B Cell	0.085514801	0.266100487
PCPG	B Cell	0.131043404	0.091407068
PRAD	B Cell	0.318508469	2.92E-11
READ	B Cell	0.072755373	0.495571724
SARC	B Cell	0.110913979	0.083813834
SKCM	B Cell	0.130131507	0.005334125
SKCM-Metastasis	B Cell	0.123750861	0.019854675
SKCM-Primary	B Cell	0.005547115	0.955873112
STAD	B Cell	0.178142862	0.000493007
TGCT	B Cell	0.26823399	0.001020509
THCA	B Cell	0.198136795	1.04E-05
THYM	B Cell	0.109307193	0.244881495
UCEC	B Cell	0.107141216	0.320433996
UCS	B Cell	0.219890107	0.113630385
UVM	B Cell	0.120139168	0.297992049
ACC	Tregs	0.020424467	0.863820702
BLCA	Tregs	-0.032060903	0.539810218
BRCA	Tregs	0.103105073	0.00113288
BRCA-Basal	Tregs	0.087830005	0.249142239
BRCA-Her2	Tregs	0.044230866	0.712184759
BRCA-LumA	Tregs	0.084440715	0.055014361
BRCA-LumB	Tregs	0.098049511	0.176054177
CESC	Tregs	0.033660606	0.576947571

CHOL	Tregs	0.275632548	0.109009062
COAD	Tregs	-0.050592077	0.403330183
DLBC	Tregs	-0.17573875	0.271742629
ESCA	Tregs	-0.237764017	0.001309307
GBM	Tregs	-0.044523878	0.605421485
HNSC	Tregs	0.098413288	0.029060815
HNSC-HPV-	Tregs	0.127141069	0.010921505
HNSC-HPV+	Tregs	0.049377241	0.645861306
KICH	Tregs	0.183992055	0.142330691
KIRC	Tregs	0.042034216	0.367875126
KIRP	Tregs	0.042342697	0.498326245
LGG	Tregs	0.236493348	1.68E-07
LIHC	Tregs	0.021069421	0.696558644
LUAD	Tregs	-0.004279905	0.924482811
LUSC	Tregs	0.013392146	0.770489584
MESO	Tregs	0.131960862	0.228636649
OV	Tregs	0.096054507	0.130639431
PAAD	Tregs	0.066521847	0.387333179
PCPG	Tregs	0.19847906	0.010132243
PRAD	Tregs	0.185738949	0.000138881
READ	Tregs	-0.016718052	0.875721462
SARC	Tregs	0.054753466	0.394478583
SKCM	Tregs	-0.02297694	0.624197342
SKCM-Metastasis	Tregs	-0.01552505	0.770985155
SKCM-Primary	Tregs	-0.136790171	0.170393616
STAD	Tregs	0.075492163	0.142399194
TGCT	Tregs	0.211210745	0.010229579
THCA	Tregs	0.194830217	1.46E-05
THYM	Tregs	0.321040086	0.000468343
UCEC	Tregs	0.028809743	0.789892347
UCS	Tregs	-0.024209597	0.863381721
UVM	Tregs	0.2282555	0.045867033
ACC	Dendritic Cell	-0.245	0.036698
BLCA	Dendritic Cell	-0.19549	0.000161
BRCA	Dendritic Cell	-0.07667	0.015613
BRCA-Basal	Dendritic Cell	-0.10494	0.16819
BRCA-Her2	Dendritic Cell	-0.21717	0.066882
BRCA-LumA	Dendritic Cell	0.022829	0.604534
BRCA-LumB	Dendritic Cell	-0.06465	0.372999
CESC	Dendritic Cell	-0.20902	0.000462
CHOL	Dendritic Cell	-0.01584	0.928019
COAD	Dendritic Cell	-0.10947	0.0699
DLBC	Dendritic Cell	-0.02864	0.858918
ESCA	Dendritic Cell	-0.06281	0.402249

GBM	Dendritic Cell	0.186098	0.029457
HNSC	Dendritic Cell	-0.03977	0.378698
HNSC-HPV-	Dendritic Cell	-0.04307	0.390268
HNSC-HPV+	Dendritic Cell	#######	0.999836
KICH	Dendritic Cell	-0.35154	0.004086
KIRC	Dendritic Cell	-0.16029	0.000551
KIRP	Dendritic Cell	-0.091	0.144954
LGG	Dendritic Cell	0.078255	0.087443
LIHC	Dendritic Cell	-0.0245	0.650141
LUAD	Dendritic Cell	-0.16797	0.000179
LUSC	Dendritic Cell	-0.14786	0.001201
MESO	Dendritic Cell	-0.24736	0.022465
OV	Dendritic Cell	-0.14752	0.01987
PAAD	Dendritic Cell	0.097725	0.20352
PCPG	Dendritic Cell	-0.16511	0.032976
PRAD	Dendritic Cell	0.037673	0.443478
READ	Dendritic Cell	-0.20646	0.050901
SARC	Dendritic Cell	-0.08428	0.189487
SKCM	Dendritic Cell	-0.13664	0.003426
SKCM-Metastasis	Dendritic Cell	-0.04617	0.386422
SKCM-Primary	Dendritic Cell	-0.07441	0.457344
STAD	Dendritic Cell	-0.11452	0.025778
TGCT	Dendritic Cell	-0.16445	0.046541
THCA	Dendritic Cell	-0.11612	0.01025
THYM	Dendritic Cell	-0.02616	0.78141
UCEC	Dendritic Cell	-0.32987	0.001698
UCS	Dendritic Cell	-0.15178	0.277959
UVM	Dendritic Cell	NA	NA
ACC	Neutrophil	0.221654	0.059478
BLCA	Neutrophil	0.096808	0.063576
BRCA	Neutrophil	0.071784	0.02362
BRCA-Basal	Neutrophil	0.001197	0.987498
BRCA-Her2	Neutrophil	0.03918	0.74385
BRCA-LumA	Neutrophil	0.067091	0.127628
BRCA-LumB	Neutrophil	0.037622	0.604398
CESC	Neutrophil	-0.04894	0.417169
CHOL	Neutrophil	-0.04318	0.805457
COAD	Neutrophil	0.107417	0.075349
DLBC	Neutrophil	-0.13538	0.398701
ESCA	Neutrophil	-0.12694	0.089479
GBM	Neutrophil	0.082524	0.337706
HNSC	Neutrophil	-0.04602	0.308345
HNSC-HPV-	Neutrophil	-0.07671	0.125592
HNSC-HPV+	Neutrophil	0.090555	0.398698

KICH	Neutrophil	0.220733	0.077238
KIRC	Neutrophil	0.160717	0.000532
KIRP	Neutrophil	0.15001	0.015888
LGG	Neutrophil	0.185025	4.70E-05
LIHC	Neutrophil	0.050709	0.347694
LUAD	Neutrophil	0.081647	0.070098
LUSC	Neutrophil	-0.01427	0.755922
MESO	Neutrophil	-0.09306	0.396931
OV	Neutrophil	0.055222	0.38558
PAAD	Neutrophil	0.033495	0.663625
PCPG	Neutrophil	-0.03535	0.650152
PRAD	Neutrophil	0.110446	0.024273
READ	Neutrophil	0.112008	0.293232
SARC	Neutrophil	0.186007	0.003544
SKCM	Neutrophil	-0.05399	0.249342
SKCM-Metastasis	Neutrophil	0.013385	0.801843
SKCM-Primary	Neutrophil	0.156231	0.116867
STAD	Neutrophil	0.042209	0.412578
TGCT	Neutrophil	-0.22281	0.006676
THCA	Neutrophil	0.099814	0.027467
THYM	Neutrophil	0.149258	0.11137
UCEC	Neutrophil	0.128974	0.231076
UCS	Neutrophil	-0.03249	0.817346
UVM	Neutrophil	-0.18073	0.115735
ACC	M1 TAMs	0.21848	0.063314
BLCA	M1 TAMs	0.085836	0.100164
BRCA	M1 TAMs	0.020847	0.511508
BRCA-Basal	M1 TAMs	0.096256	0.206413
BRCA-Her2	M1 TAMs	0.00839	0.944233
BRCA-LumA	M1 TAMs	-0.02488	0.572386
BRCA-LumB	M1 TAMs	-0.04125	0.569932
CESC	M1 TAMs	0.356731	9.80E-10
CHOL	M1 TAMs	0.044407	0.800038
COAD	M1 TAMs	0.060697	0.315918
DLBC	M1 TAMs	0.203032	0.202968
ESCA	M1 TAMs	0.0815	0.27676
GBM	M1 TAMs	0.069491	0.419726
HNSC	M1 TAMs	-0.07203	0.11053
HNSC-HPV-	M1 TAMs	-0.06916	0.167451
HNSC-HPV+	M1 TAMs	-0.0787	0.463495
KICH	M1 TAMs	0.290743	0.018795
KIRC	M1 TAMs	0.053639	0.250396
KIRP	M1 TAMs	0.109879	0.078122
LGG	M1 TAMs	0.143964	0.001601

LIHC	M1 TAMs	-0.01468	0.785922
LUAD	M1 TAMs	0.117289	0.009144
LUSC	M1 TAMs	0.072641	0.113095
MESO	M1 TAMs	0.066353	0.546276
OV	M1 TAMs	0.16701	0.008274
PAAD	M1 TAMs	-0.0472	0.539867
PCPG	M1 TAMs	0.147331	0.057431
PRAD	M1 TAMs	0.125659	0.010305
READ	M1 TAMs	0.154029	0.147202
SARC	M1 TAMs	0.196639	0.002029
SKCM	M1 TAMs	0.270121	4.41E-09
SKCM-Metastasis	M1 TAMs	0.232939	9.50E-06
SKCM-Primary	M1 TAMs	0.235432	0.017217
STAD	M1 TAMs	0.032184	0.532198
TGCT	M1 TAMs	0.128246	0.121617
THCA	M1 TAMs	0.276551	5.13E-10
THYM	M1 TAMs	-0.36209	7.00E-05
UCEC	M1 TAMs	0.374938	0.000319
UCS	M1 TAMs	0.312593	0.022674
UVM	M1 TAMs	0.377219	0.00072
ACC	M2 TAMs	0.385532	0.000756
BLCA	M2 TAMs	0.072799	0.163431
BRCA	M2 TAMs	0.119112	0.000167
BRCA-Basal	M2 TAMs	-0.05005	0.511952
BRCA-Her2	M2 TAMs	0.109419	0.360217
**BRCA-LumA**	**M2 TAMs**	**0.14664**	**0.000825**
BRCA-LumB	M2 TAMs	0.097852	0.176929
CESC	M2 TAMs	0.310754	1.29E-07
CHOL	M2 TAMs	-0.11805	0.499436
COAD	M2 TAMs	0.181318	0.002543
DLBC	M2 TAMs	-0.15999	0.317696
ESCA	M2 TAMs	0.128624	0.085286
GBM	M2 TAMs	0.200212	0.018989
HNSC	M2 TAMs	0.11682	0.009501
HNSC-HPV-	M2 TAMs	0.095514	0.056306
HNSC-HPV+	M2 TAMs	0.199066	0.061456
KICH	M2 TAMs	0.456323	0.000133
KIRC	M2 TAMs	0.154618	0.000866
KIRP	M2 TAMs	0.359029	2.88E-09
LGG	M2 TAMs	0.498122	2.43E-31
LIHC	M2 TAMs	0.097081	0.071718
LUAD	M2 TAMs	0.204453	4.73E-06
LUSC	M2 TAMs	0.233455	2.51E-07
MESO	M2 TAMs	-0.00547	0.960371

OV	M2 TAMs	0.191875	0.002359
PAAD	M2 TAMs	-0.01314	0.864591
PCPG	M2 TAMs	0.213969	0.005494
PRAD	M2 TAMs	0.196987	5.22E-05
READ	M2 TAMs	0.178494	0.092334
SARC	M2 TAMs	0.344474	3.33E-08
SKCM	M2 TAMs	0.049322	0.292738
SKCM-Metastasis	M2 TAMs	0.054337	0.307978
SKCM-Primary	M2 TAMs	0.209992	0.034145
STAD	M2 TAMs	0.066512	0.196354
TGCT	M2 TAMs	-0.13392	0.105864
THCA	M2 TAMs	-0.06061	0.181321
THYM	M2 TAMs	-0.41634	3.70E-06
UCEC	M2 TAMs	0.145441	0.176358
UCS	M2 TAMs	0.423163	0.001594
UVM	M2 TAMs	-0.21907	0.055592
