# Supplementary material for: ARL11 correlates with the immunosuppression and poor prognosis in breast cancer: A comprehensive bioinformatics analysis of ARL family members
Source: PLoS One. 2022 Nov 11;17(11):e0274757. doi: 10.1371/journal.pone.0274757 (PMC9651578; doi:10.1371/journal.pone.0274757)
Supplement: Attachment — Submitted filename: response letter.docx [file pone.0274757.s010.docx]

**Dear editors,**

**We firstly thank all the reviewers for their thorough reading of our manuscript and for the constructive suggestions/criticisms. Our point-by-point responses and the corresponding changes to the manuscript are described below.**

1. In the introduction, the statement “Breast cancer (BC) is one of the most common malignancies” could not give enough information on the morbidity of breast cancer.

**Response:** **The points raised by this reviewer are well taken and this statement was corrected in revised manuscript. We revised this sentence as “****Breast cancer is the most common malignancy in women, and one of the three most common diagnosed cancers worldwide, along with lung and colon cancer.”.**

2. Also, in the introduction, the authors should give more details on the characteristics, regulation and functions of ARLs.

**Response: In line with the reviewer’s suggestion, we gave more detailed information on ARLs (ADP-ribosylation factor-like subfamily members) in the Introduction section.**

3. In the third paragraph of discussion, the verb tense of last sentence is wrong. The authors should carefully recheck the whole paper to avoid such mistakes before publication.

**Response: Thanks for your careful review. We corrected the verb tense errors in revised manuscript.**

**Finally, we thank again you and the reviewers for your great efforts on improving the quality of this manuscript. We are looking forward to hearing your decision soon.**

**Thank you very much!**

**Best wishes,**

**Yours sincerely,**

**Yahua Song**
