# Supplementary material for: ARL11 correlates with the immunosuppression and poor prognosis in breast cancer: A comprehensive bioinformatics analysis of ARL family members
Source: PLoS One. 2022 Nov 11;17(11):e0274757. doi: 10.1371/journal.pone.0274757 (PMC9651578; doi:10.1371/journal.pone.0274757)
Supplement: Attachment — Submitted filename: response letter.docx [file pone.0274757.s012.docx]

**Dear editors,**

**We firstly thank all the reviewers for their thorough reading of our manuscript and for the constructive suggestions/criticisms. Our point-by-point responses and the corresponding changes to the manuscript are described below.**

**Reviewer #1:**

1. Introduction: Last Paragraph, the First sentence, “To date, we initially synthetically, the use of word ‘synthetically’ is not appropriate, because authors have not synthesized anything.

**Response: Thanks for your careful review. We have replaced this word in last paragraph,	Introduction section.**

2. Methods Section: First Paragraph, the First sentence, “The use of synonym ‘Synthetical and co-active’ for bioinformatic tools is also not appropriate.

**Response: Thank you. We have corrected this inappropriate sentence into “UALCAN is a comprehensive, user-friendly, and interactive web-portal”.**

3. Results: first paragraph, 4th sentence: Authors needs to confirm whether normal samples used in the analysis are ‘adjacent’ tissues from cancer patients or are they taken from normal patients with benign disease.

**Response: Thanks for your careful review. The description of “adjacent normal tissues” is not accurate and we have corrected this error in revised manuscript.**

4. Sub-heading of Results- Genetic alterations of ARLs in BC “In order to synthetically seize the expression profiles……”. Here use of the words “synthetically seize” are not appropriate.

**Response:** **Thanks for your great advice. We have changed those inappropriate statements in revised version of manuscript.**

5. Sub-heading of Results- The prognostic values of ARLs in BC “We ulteriorly explored……”. Here use of the word “ulteriorly” is not appropriate.

**Response: Thank you. We have corrected “ulteriorly” into “****furtherly”.**

6. In Figure 6 A, Authors have shown association of ARL11 expression with immune infiltration. “As shown in Fig 6A, we discovered that high ARL11 expression positively involved in the infiltration levels of CD4+ T cells (R = 0.084, P = 8.10E-03), B cells (R = 0.127, P = 6.04E-05), regulatory T cells (Tregs) (R = 0.103, P = 1.13E-03), neutrophil cells (R = 0.072, P = 2.36E-02) and M2 tumor-associated macrophages (TAMs) (R = 0.119, P = 1.67E-04). While, high ARL11 expression negatively associated with the infiltration levels of dendritic cells (DCs) (R = -0.077, P = 1.56E- 02).” In this analysis correlation coefficient is less than 0.2. Any correlation coefficient less than +0.2 or -0.2 is very weak and I mention previously these kinds of statistical associations have no practical relevance. Therefore, this paragraph and figure should be removed from the manuscript.

**Response: Thanks for your suggestion. We have removed this paragraph and related-figure in the revised manuscript.**

7. Quality of Figures is better than previous version, however the Figures are still not of publication quality. For example, Panels of Figure 5A, %C and 5D are not legible. Labels on X-axis for Figure 4A are also not legible.

**Response: Thank you very much! We have improved the quality of figures in revised version of manuscript.**

8. Several non-scientific words such as “ecumenically”, “Synthetical and co-active”, synthetically seize “Ulteriorly snatch”, “scavenge the mechanism” etc. have been used throughout the manuscript. Therefore, the manuscript needs to be proofread by a professional service provider.

**Response: Thanks for your careful review. We have corrected those inappropriate statements in revised version of manuscript.**

**Reviewer #2:**

1. The overall survival in TIMER2.0 is not a confirmation of the relapse-free survival in KM plotter, because overall survival and relapse-free survival are not the same.

**Response: Thanks for your great advice. In line with your valuable opinion, the overall survival analysis in TIMER2.0 is not a confirmation for Relapse-free survival in KM plotter in our study. We performed OS analysis to comprehensively explore the clinical values and found only ARL11 had significant prognostic values in both TCGA and GEO datasets.**

2. The conclusions from figure 6 should be toned down (Fig6A) and they appear incorrect in Fig6B (Fig6B Macrophage M2). First, even though immune infiltrates significantly correlate with ARL11 expression, the R values in Fig 6A are close to zero, and therefore an increase in ARL11 expression would result in a very small increase of immune infiltrates. Second, the data in Fig6B do not support that high ARL11 associates with shorter survival, they only support that high M2-TAMs associate with shorter survival. The significant effect is only due to the level of high M2-TAMs, which can be seen in the comparison shown in the figure between: 2 vs 1, which compares low ARL11 + high M2 (light blue line) with low ARL11 + low M2 (dark blue line), and 4 vs 3, which compares high ARL11 + high M2 (red line) with high ARL11 + low M2 (yellow line).

**Response: Thanks a lot for your suggestion. Although we found the significant relevance between ARL11-related genes and immune response in our enrichment analysis, the correlation coefficients between ARL11 expression and immune infiltrates are** **less than +0.2. We speculate that ARL11 might regulate the immune processes through other unknown mechanisms, and we would like to validate the correlation between ARL11 expression and immune processes through biological experiments in future. Thus, we decide to remove the figure 6 in revised version of manuscript.**

3. The expression values of the ARLs were evaluated in TIMER 2.0. Please include a sentence to inform the reader whether the results were the same in UALCAN and TIMER, i.e. higher in tumors than normal or vice versa.

**Response: Thanks for your careful review. We have compared the expression status of ARLs in UALCAN and TIMER databases and added related-paragraphs** **into the end of the Result 1 and added more expression details in S1 Table in revised version.**

4. According to the manuscript, the methylation status of ARL4A is supposed to be seen in Table 1, but there is only ARL4C and ARL4D.

**Response: Thanks for your careful review. In this study, we analyzed the relationship between the status of promoter DNA methylation with levels of ARLs mRNA expression. Among 22 ARLs, the correlation coefficient between mRNA expression and DNA methylation of ARL3, ARL4C, ARL4D and ARL11 were relatively higher (R ≥ 0.5, P < 0.05) . Therefore, we have shown the specific methylation site of ARL3, ARL4C, ARL4D and ARL11 in Table 1. In previous version of manuscript, we make some clerical errors, and we have corrected it in revised version.**

5. „…..copy number amplification and DNA methylation might be involved in the epigenetic regulation of ARLs.“ Copy number amplification (CNA) is not considered an epigenetic mechanism. However, CNA may well regulate ARL expression levels.

**Response: Thanks. Sorry for this inaccurate statement, we have corrected this point in revised manuscript.**

6. The figure legends can be more detailed in many cases. For example, Fig 3 is completely missing a legend, Fig 4 would be easier to understand if more explanations were included, and in Fig 6 there is not a positive correlation with CD8+ T cells or M1-TAMs. Please amend.

**Response: Thanks for your valuable suggestion. We have added more detailed information into figure legends.**

7. They are called Circos plots with ´o´ not Circus with ´u´.

**Response: Thanks for your suggestion. We have corrected these errors.**

8. The text has been changed for the worse in the tracked version of the manuscript. The original text was more appropriate in many instances. Please fix.

**Response: Thanks for your great advice. We have changed those inappropriate statements in revised version of manuscript.**

**Finally, we thank you and the reviewers again for your great efforts on improving the quality of this manuscript. We are looking forward to hearing your decision soon.**

**Thank you very much!**

**Best wishes,**

**Yours sincerely,**

**Yahua Song**
