# Supplementary material for: ARL11 correlates with the immunosuppression and poor prognosis in breast cancer: A comprehensive bioinformatics analysis of ARL family members
Source: PLoS One. 2022 Nov 11;17(11):e0274757. doi: 10.1371/journal.pone.0274757 (PMC9651578; doi:10.1371/journal.pone.0274757)
Supplement: Attachment — Submitted filename: response letter.docx [file pone.0274757.s013.docx]

**Dear editors,**

**We firstly thank all the reviewers for their thorough reading of our manuscript and for the constructive suggestions/criticisms.**

**In line with your suggestion, the revised version of manuscript has been performed professional English language editing and review services by AiMi Academic Services (www.aimieditor.com).**

**Finally, we thank you and the reviewers again for your great efforts on improving the quality of this manuscript. We are looking forward to hearing your decision soon.**

**Thank you very much!**

**Best wishes,**

**Yours sincerely,**

**Yahua Song**
